# A Case of Successful Biventricular Repair of the Transposition of the Great Arteries with a Coronary Anomaly Associated with an Atrioventricular Septal Defect

**DOI:** 10.1007/s00246-023-03276-w

**Published:** 2023-08-25

**Authors:** Hisayuki Hongu, Koji Nomura, Izumi Hamaya, Shinya Ugaki, Toshikazu Shimizu, Makiko Nisioka, Kenji Hoshino

**Affiliations:** 1 Department of Cardiovascular Surgery, Saitama Children’s Medical Center, Zip 330-8777, 1-2 Shintoshin, Chuo-Ku, Saitama, 330-8777 Japan; 2 Department of Cardiology, Saitama Children’s Medical Center, Saitama, Japan

**Keywords:** Atrioventricular septal defect, Transposition of the great arteries, Modified one-patch method, Lecompte maneuver

## Abstract

The transposition of the great arteries (TGA) associated with a complete atrioventricular septal defect is a rare and serious congenital cardiac anomaly. In this report, we describe the successful biventricular repair of a TGA with a complete atrioventricular septal defect in an infant. Due to the low body weight of the patient and a complex coronary pattern anomaly, an arterial switch operation was executed, with the Mee procedure and pulmonary arterial banding as initial palliative measures when the infant was 22 days old and weighed 2.5 kg. Subsequently, atrioventricular septal defect repair using the modified one-patch method was performed when the patient was 1.3 years old and weighed 8.8 kg. Remarkably, the postoperative course of the patient demonstrated no notable incidents. To our knowledge, this is the first time a two-stage strategy was applied to repair these complex defects, presenting a promising approach for managing similar cases in future medical practice.

## Case Report

**Fig. 1:**
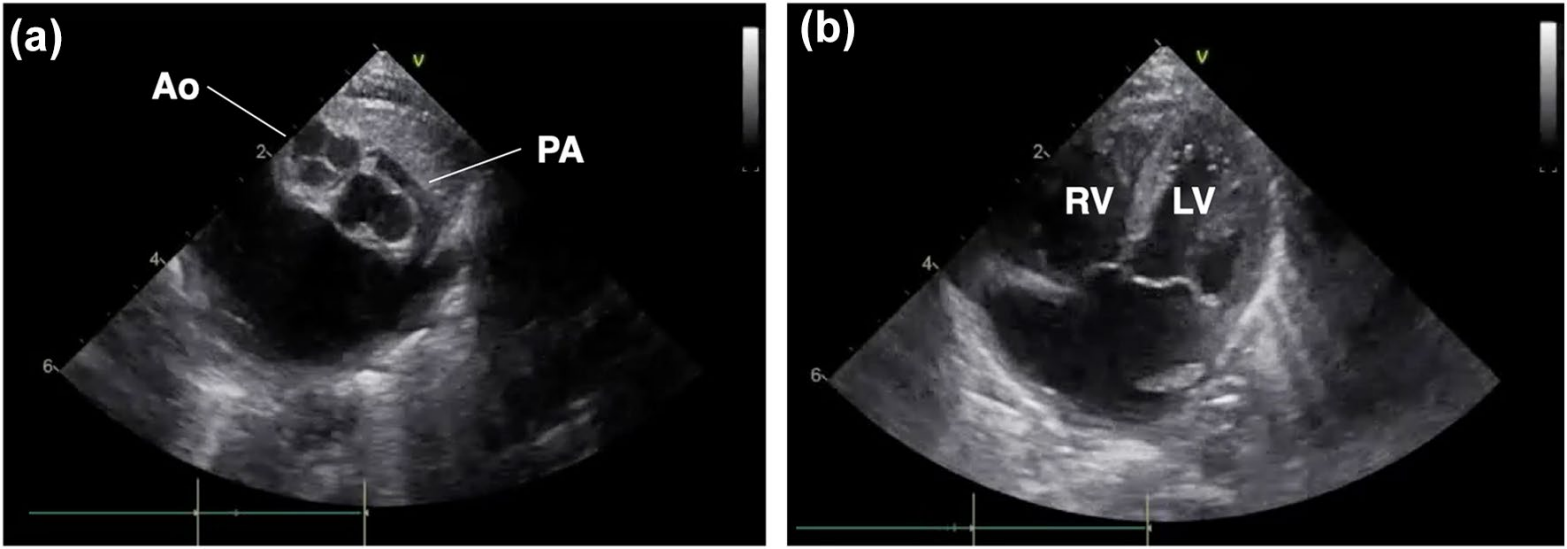
Preoperative echocardiography imaging. **a** The aorta was positioned right anterior, and the pulmonary artery was positioned left posterior; *Ao* aorta, *PA* pulmonary artery. **b** Four-chamber view showing balanced ventricles; *LV* left ventricle, *RV* right ventricle

A female neonate with a postnatal diagnosis of complete transposition of the great arteries (TGA) and Rastelli A-type complete atrioventricular septal defect (AVSD) was referred to our institution. Cardiac catheterization and echocardiography revealed an anteroposterior relationship between the great arteries (right anterior aorta and left posterior pulmonary artery) (Fig. 1a). An echocardiography-based preoperative diagnosis of the coronary artery pattern determined that the neonate had a Shaher type 2A classification, with the left anterior descending branch (LAD) originating from sinus 1 and the common trunk of the left circumflex branch (LCx) and right coronary artery (RCA) originating from sinus 2. Both ventricles had balanced volumes (Fig. 1b) and good function.

**Fig. 2:**
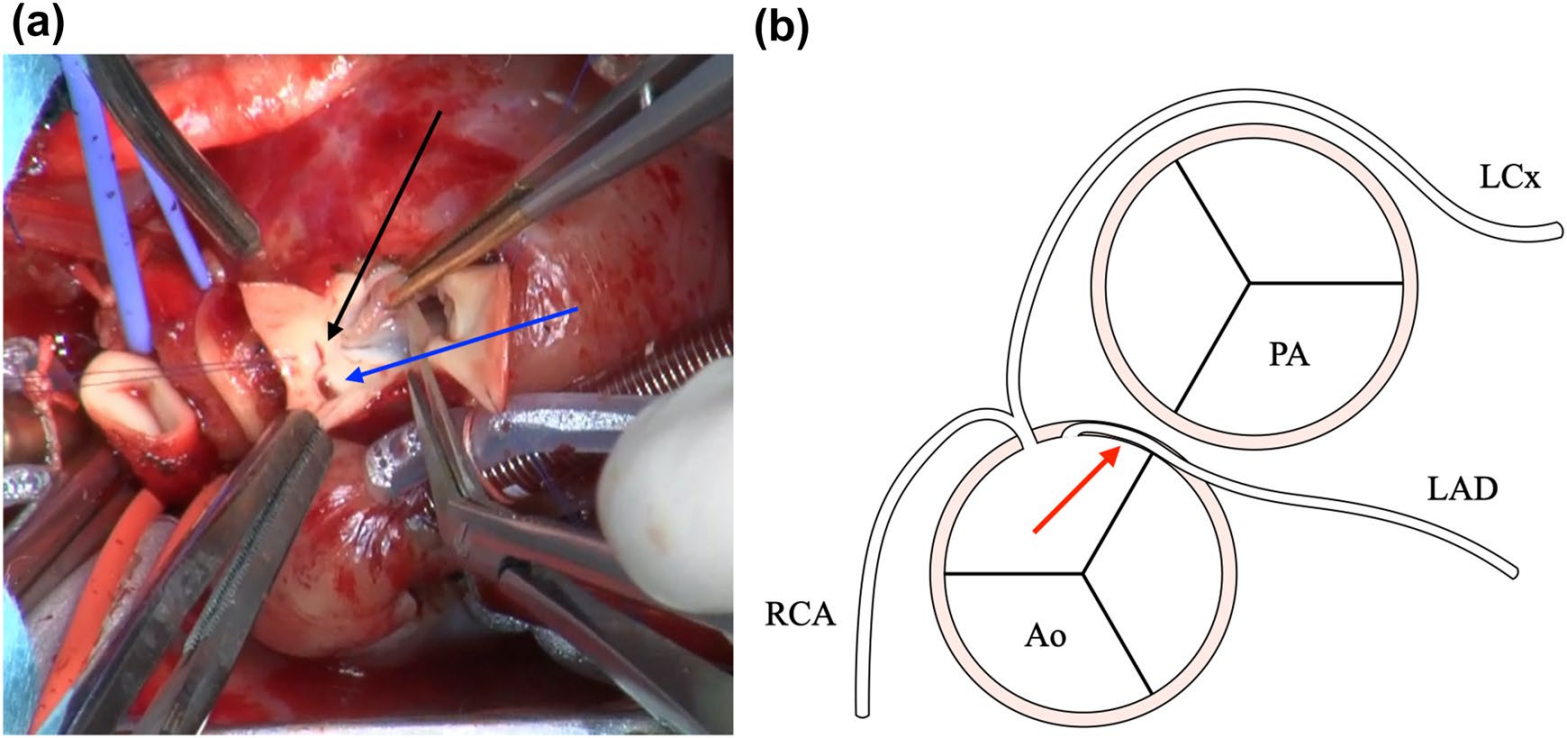
**a** Intra-operative findings of coronary arterial origins during the first palliative procedure. The black arrow shows the orifice of the left anterior descending branch, and the blue arrow shows the orifices of the left circumflex branch and right coronary artery. **b** Schematic representation of the relationship between the great arteries and coronary arteries. The red arrow represents the intramural region of the left anterior descending branch. *Ao* aorta, *PA* pulmonary artery, *RCA* right coronary artery, *LAD* left anterior descending branch, *LCx* left circumflex branch

**Fig. 3:**
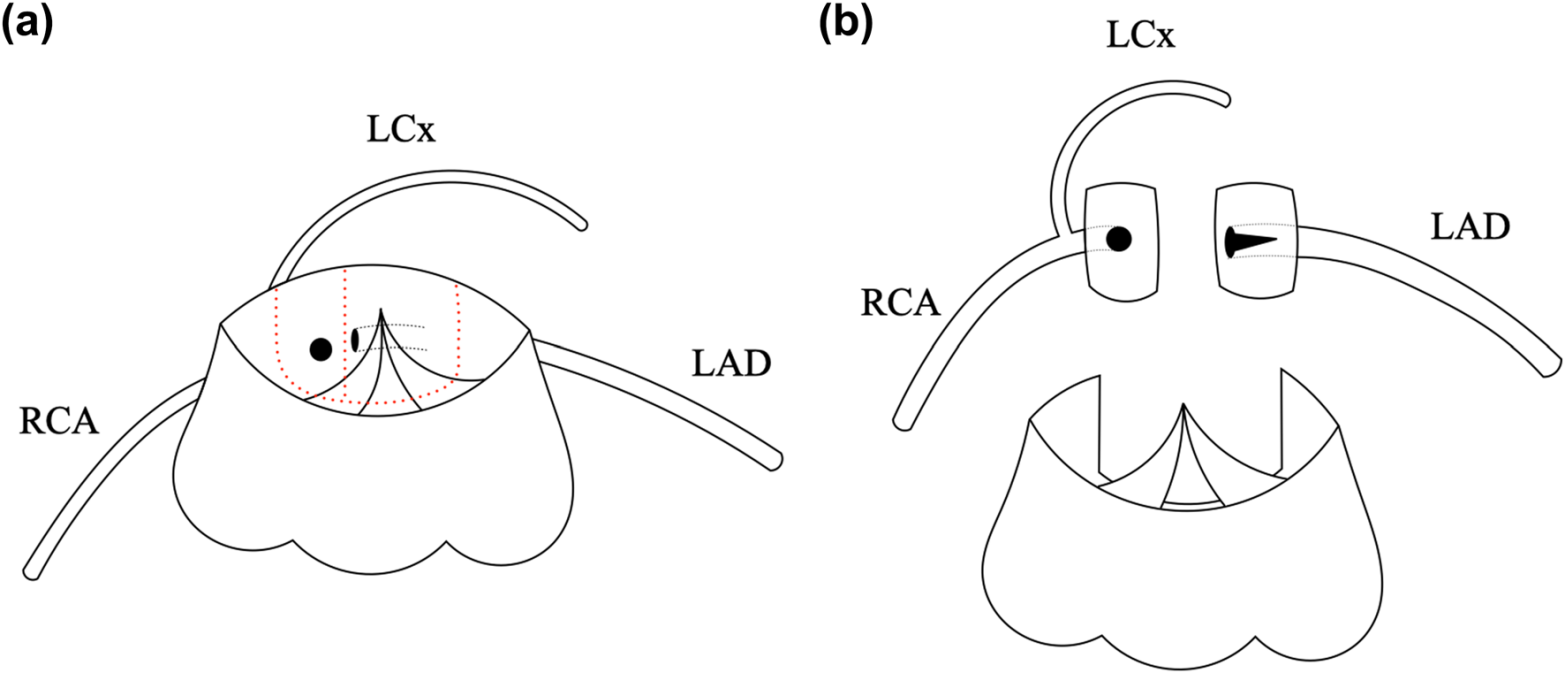
Schematic representation of the coronary artery re-implantation during the arterial switch operation. **a** The schema displays the origins of the coronary artery, and the red dotted line represents the incision line of coronary cuffs. **b** Each coronary cuff is divided, and the orifice of the left anterior descending branch is incised and enlarged to unroof the intramural segment. *RCA* right coronary artery, *LAD* left anterior descending branch, *LCx* left circumflex branch

Because of the infant’s low body weight, a single-stage complete repair was deemed too difficult; therefore, a staged approach was selected. As initial palliative measures, an arterial switch operation (ASO) and main pulmonary artery (PA) banding were performed via median sternotomy when the neonate was 22 days old and weighed 2.5 kg. Accordingly, a cardiopulmonary bypass was established using aortic and bicaval cannulations. A search for coronary arterial origins showed that the orifices of the RCA and LCx originated from sinus 2, but the location of the LAD orifice as visualized from outside the aorta was not apparent, suggesting that the LAD ran an intramural course. After initiating cardioplegic arrest, the ascending aorta was transected, and the orifice location of each coronary artery was accurately identified (Fig. 2). Both coronary orifices originated from sinus 2 and were close together; therefore, the correct diagnosis was atypical Shaher type 5. The LAD orifice was incised and enlarged to unroof the intramural segment. The posterior commissure was detached from the aortic wall. Both coronary orifices were resected as one large cuff, as these could barely be divided into two separate cuffs and were then re-implanted into the neo-aortic root according to the Mee procedure (Fig. 3) [1]. PA reconstruction was performed using the LeCompte maneuver [2]. Withdrawal from cardiopulmonary bypass was uneventful. After decannulation, PA banding was performed on the arterial circumference (body weight + 19.5 mm). Postoperative hemodynamics were stable, but the PA banding was removed following delayed sternal closure due to mild peripheral PA stenosis.

Two months after the ASO, cardiac catheterization indicated that the left ventricle (LV)/right ventricle (RV) end-diastolic volume was 118%/90% of normal. The LV/RV ejection fractions were 61% and 68%, respectively. In addition, the (mean) PA pressure was 29/7 mmHg (12 mmHg).

**Fig. 4:**
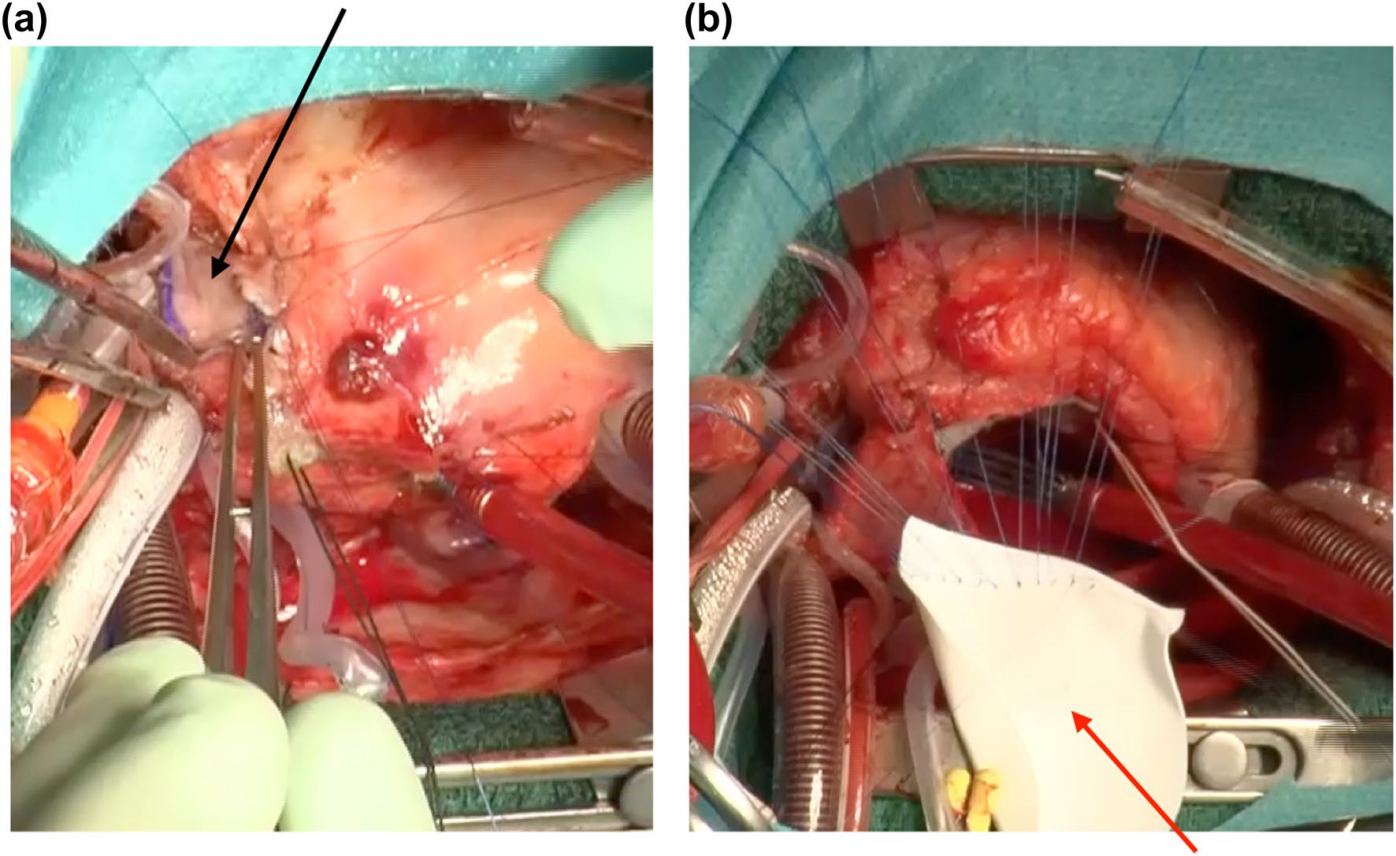
Intra-operative findings during the second procedure to completely repair the atrioventricular septal defect. **a** Pulmonary artery augmentation was performed with an autologous pericardium patch (black arrow). **b** The atrioventricular septal defect was repaired using the modified one-patch method. The sutures on the edge of the ventricular septal defect were placed through the division line of the common atrioventricular valve to the expanded polytetrafluoroethylene patch (blue arrow) used for the atrial septal defect

The patient’s body weight increased sufficiently; subsequently, AVSD repair was performed as an intracardiac procedure once the patient turned 1.3 years old and weighed 8.8 kg. Concomitantly, PA augmentation using an autologous pericardial patch was performed to address the PA bifurcation stenosis (Fig. 4a). As the ventricular septal defect was small (approximately 3 mm), the AVSD was repaired using a modified one-patch procedure [3]. The primary atrial septal defect was closed using an expanded polytetrafluoroethylene patch (Fig. 4b).

The patient’s postoperative course was favorable; she was extubated on postoperative day 1 and discharged on postoperative day 17. Echocardiography performed on postoperative day 14 demonstrated an ejection fraction of 79%, and LV outflow tract obstruction was not observed. Right/left atrioventricular valve regurgitation was mild-to-moderate/none, respectively. The maximum velocity in the right/left PA was measured at 2.6 m/s/1.8 m/s, respectively. The infant is currently a healthy outpatient with no symptoms of heart failure.

## Discussion

TGA with AVSD is a rare congenital cardiac anomaly. In an autopsy series of 507 patients with AVSD, Bharati et al. found only 17 TGA cases [4]. In such a condition, many patients have unbalanced ventricles and may be potential candidates for the Fontan procedure [5]. Even if a patient has balanced ventricles, many have LV outflow tract obstruction and/or atresia [6]. This condition is usually treated using the Rastelli procedure. In this case, no LV outflow tract obstruction was observed. Therefore, any surgical intervention had to be considered within the first two weeks of life when the LV pressure has decreased due to improvements in neonatal physiological pulmonary hypertension after cardiopulmonary transition at birth.

Currently, the surgical outcomes of ASO for TGA are improving, and it is considered the first choice in many cases as a relatively safe procedure. However, AVSD repair in the neonatal period is more technically challenging because of the delicate intricacy of the atrioventricular valve. Victor et al. reported four cases of such complex defects resolved through neonatal single-stage complete repair [7]. Considering the outcomes of the procedure, one of the four patients died in the hospital and one patient required reoperation in the late phase, this single-stage strategy may not be the most appropriate approach for our patient. In our case, a one-stage complete repair may have been challenging because of the low body weight and complex coronary artery pattern; therefore, a staged repair was considered preferable.

Some reports have shown good results for the staged repair of this complex. Almost all reports present neonatal PA banding prior to ASO, AVSD repair, and PA de-banding in late infancy [8, 9]. The merit of this strategy is the improvement of heart failure symptoms and/or maintenance of LV pressure without the use of cardiopulmonary bypass in the neonatal period. However, there are some problems with this approach, including technical difficulties encountered during coronary artery dissection due to adhesions, re-sternotomy conditions, or degeneration of the neo-aortic valve after PA banding. In our approach, cardiopulmonary bypass in the neonatal period was certainly a disadvantage; however, the patient’s general condition post procedure was acceptable. To the best of our knowledge, this is the first time this staged strategy was applied to this complex.

In conclusion, ASO in the neonatal period followed by AVSD repair in late infancy is a useful and safe multi-stage strategy for approaching TGA and AVSD, despite some challenges, such as low body weight or anomalous coronary patterns.

## Abbreviations

*TGA*: Complete transposition of the great arteries
*AVSD*: Complete atrioventricular septal defect
*LAD*: Left anterior descending branch
*LCx*: Left circumflex branch
*RCA*: And right coronary artery
*PA*: Pulmonary artery
*LV*: Left ventricle
*RV*: Right ventricle
